# Trends in use of Attention-Deficit Hyperactivity Disorder medications among children and adults in five European countries, 2010 to 2023: a population-based observational study

**DOI:** 10.1016/j.lanepe.2025.101556

**Published:** 2026-01-21

**Authors:** Xintong Li, Yuchen Guo, Agustina Giuliodori Picco, Anna Palomar-Cros, Antonella Delmestri, Wai Yi Man, Isabella Kaczmarczyk, James T. Brash, Katia Verhamme, Mees Mosseveld, Talita Duarte-Salles, Daniel Prieto-Alhambra, Edward Burn

**Affiliations:** a Pharmaco- and Device Epidemiology, Health Data Sciences, Nuffield Department of Orthopaedics, Rheumatology and Musculoskeletal Sciences, University of Oxford, United Kingdom; b Fundació Institut Universitari per a la recerca a l'Atenció Primària de Salut Jordi Gol i Gurina (IDIAPJGol), Spain; c Data Strategy, Access and Enablement (DSAE), IQVIA, United Kingdom; d Department of Medical Informatics, Erasmus University Medical Center, the Netherlands

**Keywords:** ADHD, Observational study, Electronic health records, Real-world evidence, Stimulants, Pharmacotherapy

## Abstract

**Background:**

An increase in the use of medications for Attention-Deficit Hyperactivity Disorder (ADHD) has been reported globally. This study aims to estimate the trends of ADHD medications use among children and adults across Europe from 2010 to 2023.

**Methods:**

We conducted a population-level observational study using electronic health records from five European countries: Belgium, Germany, the Netherlands, Spain, and the UK. We estimated the prevalence and incidence of methylphenidate, dexamphetamine, lisdexamfetamine, atomoxetine and guanfacine use among individuals aged 3 years and older. We used the proportion of patients covered to measure treatment adherence. All analyses were reported by country and stratified by age group and sex.

**Findings:**

The prevalence of ADHD medication use increased across all five countries during the study period. Between 2010 and 2023, prevalence rose more than threefold in the UK (from 0.12% to 0.39%) and more than doubled in the Netherlands (from 0.67% to 1.56%). Adult use increased substantially in all countries, particularly among females. In the UK, prevalence among adults aged over 25 increased from 0.01% in 2010 to approximately 0.20% in 2023, representing a more than twenty-fold increase in females and fifteen-fold in males. Although ADHD medication use remained higher among males, the sex gap in treatment narrowed over time and with increasing age. After 1-year of medication initiation, 14.9%, 16.0%, 43.9%, and 30.8% of participants were covered by treatment in Germany, the Netherlands, Spain, and the UK respectively. Among initiators, the prevalence of psychiatric conditions and prior use of psycholeptic medications was higher in females and in older age groups.

**Interpretation:**

Over 14 years, ADHD medication prevalence increased across Europe, with varying incidence trends by country, age, and sex. Understanding the utilisation of ADHD medications can provide useful information in monitoring use, as well as for anticipation and planning to minimise potential shortages.

**Funding:**

10.13039/501100013120European Medicines Agency.

**Research in context**

**Evidence before this study**

Attention-Deficit Hyperactivity Disorder (ADHD) diagnoses have increased globally over the past few decades, and increase in use of ADHD medications have been reported globally. To assess available evidence of the trends in ADHD medication use in real-world settings, we searched PubMed and Google Scholar for studies written in English, published from January 1, 2010, to September 10, 2025, using the following terms in all fields with no restrictions on publication type: (“treatment” OR “medication” OR “medicine”) AND (“prevalence” OR “incidence” OR “trend” OR “utilisation” OR “utilization” OR “consumption”) AND (“attention-deficit hyperactivity disorder” OR “ADHD” OR “hyperkinetic disorder”) AND (“observational” OR “population-based” OR “real-world”). The search yielded 482 articles. We screened the articles by their titles and reviewed the abstracts of the remaining articles to identify potentially relevant studies. Additionally, we scanned the reference lists of relevant articles. The inclusion criteria required that the study reported ADHD medication use in the general population using observational data.

Most of the studies we found were from Europe or North America, typically using older data, especially from before the COVID-19 pandemic. These studies often focused on children rather than adults, included only a single database, and primarily estimated prevalence due to the lack of reliable denominator estimates. We identified seven multinational/multi database studies: three from Scandinavian countries, one included four European databases and US data 2005–2012 focused on children and adolescents, one using 13 population-based databases from 2001 to 2015, and two evaluating medication consumption using pharmaceutical sales data. However, differences in study designs make direct comparison of the estimates challenging.

**Added value of this study**

In this multinational observational study, we utilised routinely collected health data from five countries within the Data Analysis and Real World Interrogation Network (DARWIN EU®) to estimate the incidence and prevalence of ADHD medications among children and adults during 2010–2023. While the trend of use among children and adolescents varied across databases, we observed that both the prevalence and incidence of ADHD medication use increased among adults in all databases, with the difference between males and females decreasing over the years. We observed that a substantial proportion of people who used ADHD medication also used antidepressants and psycholeptics, especially among adults.

**Implications of all the available evidence**

Understanding the utilisation of ADHD medications can provide useful information in monitoring use, as well as for anticipation and planning to minimise potential shortages. The high prevalence of comorbidities and other medication use among initiators also highlights the importance of further understanding of the safety and effectiveness of these medications, as these populations are usually excluded from clinical trials.

## Introduction

Attention-deficit hyperactivity disorder (ADHD) is a prevalent neurodevelopmental disorder characterized by symptoms of inattention, hyperactivity, and impulsivity. Globally, the prevalence of ADHD is estimated at 8% in children and adolescents and 3% in adults.^1^^,^^2^ ADHD diagnoses have increased globally over the past few decades, including in Europe. Pharmacological treatment plays a central role in symptom management and improving functional outcomes. Over the last two decades, new medications have been introduced for the management of ADHD, and the indications for existing drugs have been expanded to include different age groups. Methylphenidate remains the suggested first-line treatment for ADHD in children and adults, due to its established efficacy and safety profile.^3^

This rise in diagnosis has been accompanied by a corresponding increase in the use of medications to treat ADHD globally,^4^, ^5^, ^6^, ^7^ raising concerns about possible overdiagnosis and inappropriate prescribing of ADHD medications. However, patterns of ADHD medication use vary widely across countries and over time, reflecting differences prescribing practices in treating people with ADHD, healthcare access, and public attitudes toward mental health and neurodevelopmental disorders.

Understanding the ADHD medication use trends over time can support evidence-based planning and resource allocation within healthcare systems, insofar as historical patterns provide insight into future needs. These data also contribute to public health surveillance by identifying population-level changes in prescribing practices and informing strategies to optimise care delivery. Since September 2023, a global shortage of ADHD medications has emerged and remains ongoing in some European countries and regions, primarily due to rising demand and manufacturing challenges.^8^ Monitoring recent usage patterns can support anticipating demands for ADHD medications and timely responses and help mitigate future shortages.

Despite growing interest in ADHD medication use, most existing studies were limited in scope. The majority were conducted in Europe or North America and relied on older data, often predating the COVID-19 pandemic. These studies typically focused on children rather than adults, used a single database, and primarily estimated prevalence due to the lack of reliable denominator data for incidence calculations. However, differences in study designs, populations, and data sources make direct comparisons of estimates challenging.

The Data Analysis and Real World Interrogation Network (DARWIN EU®) is an initiative founded by the European Medicines Agency (EMA) to provide timely and reliable evidence on the use, safety and effectiveness of medicines using real world healthcare databases across the European Union (EU).^9^ Through DARWIN EU®, the current study was requested by EMA to support evidence-based planning of ADHD medications. By providing harmonised, multi-country estimates of ADHD medication use, the study aims to provide updated trends in the prevalence (the proportion of the population using a medication) and incidence (the number of new users of a medication per population over a specified time period) of ADHD medications use in children and adults across Europe. It also seeks to determine the current evidence on the utilisation and adherence of ADHD medications during this period.

## Methods

### Study design and data source

We conducted a multinational observational study using population-based routinely collected electronic health records data from five European countries within the DARWIN EU® network: Belgium (IQVIA Longitudinal Patient Data Belgium, IQVIA LPD Belgium), Germany (IQVIA Disease Analyzer Germany, IQVIA DA Germany), the Netherlands (Integrated Primary Care Information Project, IPCI), Spain (the Information System for the Development of Research in Primary Care, SIDIAP), and the UK (Clinical Practice Research Datalink, CPRD GOLD).

The Belgian data include records from general practices, with information on medication use available from 2015 onwards. The German data encompass both general primary care and specialised practices. Data from the Netherlands and the UK are derived from primary care settings, though they contain limited information on hospital admissions. The Spanish data contain primary care records, with linkage to hospital information. Additional detailed description of each database is available in Appendix 1.

The study was conducted using a common data model approach, where different data sources are transferred into a standard data structure, facilitating the analysis of healthcare data from multiple databases. In DARWIN EU®, all data partners had mapped their data into the Observational Medical Outcomes Partnership Common Data Model (OMOP CDM).^10^ The OMOP CDM enabled the same analytical code to be executed across multiple data sources in a federated manner, without transferring or directly sharing the underlying individual-level data.

The study period began on 1 January 2010 and ended in mid to late 2023, depending on data availability for each participating database. For the Belgian data, the study period started in 2015. This timeframe allowed us to assess ADHD medication use before and after key clinical and policy changes, including the revision of ADHD diagnostic criteria in DSM-5 (2013). It also enabled comparison with previous studies, ensured sufficient data to capture trends leading up to these changes, and provided contemporary data on prescribing trends.

### Study population and study medication

The study population included all individuals aged three years and older who were registered in the respective databases during the study period. We also required individuals to have at least 365 days of prior data availability. We excluded individuals with missing data on sex or age. The study population was stratified into four age groups: children (aged 3–11 years), adolescents (12–17 years), young adults (18–24 years), and adults (≥25 years). These age groups were defined based on differences in diagnostic criteria and clinical management of ADHD, the approved indications for the study medications, and to ensure consistency with other multi-database studies.^11^

ADHD medications were identified using WHO Anatomical Therapeutic Chemical classification codes. We included five medications licensed for ADHD treatment within Europe: methylphenidate (N06BA04), dexamphetamine (N06BA02), lisdexamfetamine (N06BA12), atomoxetine (N06BA09) and guanfacine (C02AC02). Exposure was defined as an ADHD medication record (either prescribed or dispensed) during the study period. We used a grace period of 30-day to define continuous treatment episode. We defined incident user using a 365-day washout window. Use of ADHD medication was examined regardless of a diagnosis of ADHD.

### Statistical analysis

We described the characteristics of individuals who initiated ADHD medication. Categorical data for participant characteristics are presented as frequencies (percentages), and medians and interquartile ranges (IQRs) for continuous variables, for people who initiated any of the ADHD medication, and stratified by medication, age group, and sex.

We calculated yearly period prevalence of ADHD medication use, which summarises the total number of users of the drug of interest during a given calendar year divided by the population under observation during that year. We did not require the denominator population to be under observation for the entire year. Binomial 95% confidence intervals have been calculated.

Yearly incidence rates of ADHD medications were calculated as the number of incident users per 100 000 person-years of the population at risk of getting exposed during each year assuming a Poisson distribution. The analyses of prevalence and incidence rates were stratified by age group and sex. We also calculated the male-to-female incidence rate ratio within each age group.

We estimated medication treatment persistence over the five-year period following initiation using the proportion of patients covered (PPC), which allows individuals to re-enter the analysis after a break in treatment. The numerator comprises individuals who are alive and currently covered by medication on a given day after initiation, while the denominator includes all individuals who initiated the medication and remained under follow-up.^12^ This method has been used to study treatment adherence and is less sensitive to changes in grace periods.^11^ Due to the lack of reliable prescription length information in the Belgium data, it did not contribute to this analysis. As a sensitivity analysis, we used a 90-day grace period to define treatment episode to account for potential drug holidays.

All statistical analyses were performed using R (version 4.2.3) through RStudio.^13^ R package “*IncidencePrevalence*” (version 1.0.0)^14^ was used for estimating of incidence and prevalence, and “DrugUtilisation” (version 0.8.2)^15^ was used for estimating PPC. Study findings are reported according to the Strengthening the Reporting of Observational Studies in Epidemiology (STROBE) statement.

### Ethical approval

The study was approved by the CPRD's Research Data Governance Process (24_004294), the Clinical Research Ethics committee of Fundació Institut Universitari per a la recerca a l’Atenció Primària de Salut Jordi Gol i Gurina (IDIAPJGol) (approval number 24/197-Eom), and the IPCI Review Board (registration no.15/2024). Ethical approval is not required from IQVIA databases.

### Role of the funding source

This study was funded by EMA and performed via DARWIN EU®. The study funder was involved in revising the study protocol and the objectives and reviewing the study report including the results. This communication represents the views of the DARWIN EU® Coordination Centre only and cannot be interpreted as reflecting those of the EMA or the European Medicines Regulatory Network.

## Results

We included a total of 198 167 individuals who initiated any approved ADHD medication across all databases during the study period of 2010–2023. (Belgium–IQVIA LPD Belgium: 4689; Germany–IQVIA DA Germany: 46414; the Netherlands–IPCI: 51796; Spain–SIDIAP: 64039; the UK–CPRD GOLD: 31229). In the Belgium data, only users of atomoxetine, guanfacine, and methylphenidate were identified. Dextroamphetamine use was not found in Spain.

Across all countries, ADHD medication initiators were predominantly male, ranging from 60% in the Netherlands to 72% in the UK (Table 1). The median age of users ranged from 14 years old in Germany and Spain to 20 years old in the Netherlands. Common comorbidities prior to medication initiation included depression (prevalence ranged from 8% in Spain to 19% in Germany), anxiety (prevalence ranged from 13% in Belgium to 24% in the Netherlands), and asthma (prevalence ranged from 7% in Germany and the Netherlands to 17% in Belgium).

**Table 1:** Characteristics of people initiating ADHD medications during the study period, by database.

Variable	Database				
	IQVIA LPD Belgium	IQVIA DA Germany	IPCI	SIDIAP	CPRD GOLD
Country/Region	Belgium	Germany	Netherlands	Catalonia, Spain	UK
Number of participants					
–	4689	46,414	51,796	64,039	31,229
Number of treatment episodes					
–	5805	52,901	61,946	76,952	34,397
Age Median [Q25–Q75]					
–	19 [13–29]	14 [10–24]	20 [13–33]	14 [10–23]	15 [10–24]
Age group, N (%)					
3–11	1024 (18%)	17,756 (34%)	12,005 (19%)	25,883 (34%)	12,025 (35%)
12–17	1556 (27%)	15,795 (30%)	14,629 (24%)	25,492 (33%)	8994 (26%)
18–24	1417 (24%)	6309 (12%)	11,537 (19%)	6957 (9%)	5193 (15%)
25 +	1808 (31%)	13,041 (25%)	23,775 (38%)	18,620 (24%)	8185 (24%)
Sex, N (%)					
Female	2074 (36%)	15,403 (29%)	24,826 (40%)	25,131 (33%)	9797 (28%)
Comorbidity any time prior, N (%)					
ADHD	4899 (84%)	44,614 (84%)	34,899 (56%)	40,312 (52%)	22,530 (65%)
Anxiety	762 (13%)	8076 (15%)	14,870 (24%)	13,233 (17%)	6751 (20%)
Asthma	999 (17%)	3673 (7%)	4357 (7%)	6268 (8%)	3737 (11%)
Autism	176 (3%)	2463 (5%)	3013 (5%)	4225 (5%)	3877 (11%)
Behavioural disorder	<5 (<5%)	12,013 (23%)	389 (1%)	7663 (10%)	965 (3%)
COPD	534 (9%)	2942 (6%)	301 (0%)	478 (1%)	54 (0%)
Depression	1027 (18%)	10,025 (19%)	5221 (8%)	6721 (9%)	3490 (10%)
Diabetes	108 (2%)	558 (1%)	784 (1%)	1193 (2%)	302 (1%)
Eating disorder	19 (0%)	1059 (2%)	454 (1%)	1307 (2%)	265 (1%)
Fatigue	337 (6%)	1540 (3%)	10,483 (17%)	4092 (5%)	1959 (6%)
Gastroesophageal reflux disease	543 (9%)	410 (1%)	417 (1%)	1466 (2%)	656 (2%)
Hypertension	311 (5%)	1101 (2%)	1523 (2%)	2216 (3%)	284 (1%)
Major depressive disorder	66 (1%)	6365 (12%)	1165 (2%)	6280 (8%)	168 (0%)
Malignancy	42 (1%)	346 (1%)	858 (1%)	2306 (3%)	175 (1%)
Mood disorders	1050 (18%)	6989 (13%)	4822 (8%)	2268 (3%)	3580 (10%)
Narcolepsy	45 (1%)	431 (1%)	0 (0%)	212 (0%)	177 (1%)
Obesity	72 (1%)	2572 (5%)	1084 (2%)	7981 (10%)	272 (1%)
Osteoarthritis	287 (5%)	728 (1%)	512 (1%)	1999 (3%)	264 (1%)
Pneumonia	181 (3%)	2290 (4%)	1938 (3%)	6657 (9%)	252 (1%)
Urinary tract infectious disease	198 (3%)	2106 (4%)	4384 (7%)	6033 (8%)	1476 (4%)
Medications use any time prior, N (%)					
Agents acting on the renin angiotensin system	141 (2%)	515 (1%)	1539 (2%)	3152 (4%)	407 (1%)
Antibacterials	3283 (57%)	12,336 (23%)	26,753 (43%)	57,431 (75%)	25,606 (74%)
Antidepressants	1078 (19%)	8512 (16%)	9014 (15%)	18,377 (24%)	8440 (25%)
Antiepileptics	314 (5%)	1734 (3%)	1711 (3%)	12,072 (16%)	2283 (7%)
Anti-inflammatory and antirheumatic products	2523 (43%)	13,087 (25%)	15,800 (26%)	60,990 (79%)	11,608 (34%)
Antithrombotic agents	68 (1%)	271 (1%)	2337 (4%)	3180 (4%)	279 (1%)
Beta blocking agents	319 (5%)	652 (1%)	2825 (5%)	2318 (3%)	2931 (9%)
Drugs for acid related disorders	1218 (21%)	2052 (4%)	10,311 (17%)	15,909 (21%)	7414 (22%)
Drugs for obstructive airway diseases	2076 (36%)	7190 (14%)	18,730 (30%)	34,182 (44%)	13,973 (41%)
Lipid modifying agents	169 (3%)	248 (0%)	1379 (2%)	3629 (5%)	389 (1%)
Opioids	866 (15%)	1462 (3%)	4967 (8%)	13,762 (18%)	4831 (14%)
Psycholeptics	1440 (25%)	7270 (14%)	16,362 (26%)	33,602 (44%)	12,682 (37%)

**Fig. 1:**
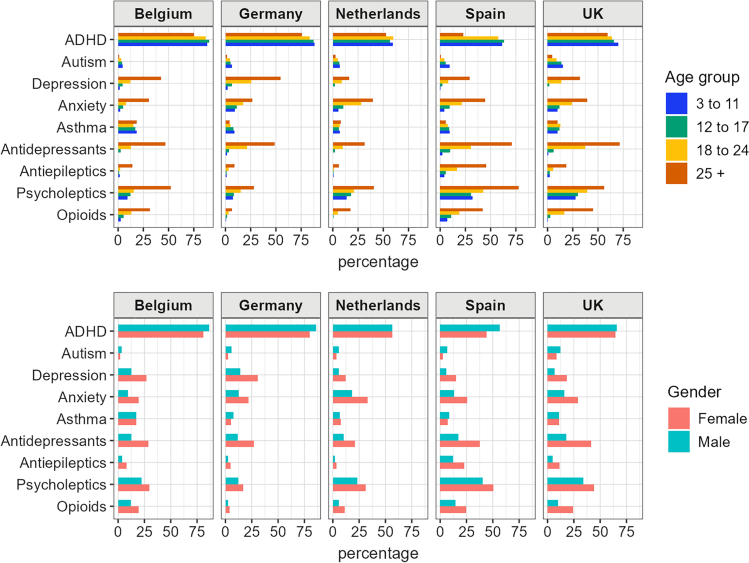
Selected comorbidities and medication use any time prior to ADHD medication use, by age group, sex and country.

Fig. 1 presents the selected comorbidities and medication use by age groups and sex. In all five databases, individuals aged 25 or over had a higher prevalence of depression and anxiety as compared to other age groups. Prior use of psycholeptics and antidepressants was higher in the older age groups and particularly common among individuals aged 25 and above. In Spain and the UK, over 70% of people aged over 25 had previously used antidepressants. The prevalence of depression and anxiety in females was approximately twice that observed in males, and males showed a higher prevalence of autism.

### Trends in prevalence of ADHD medication use

**Fig. 2:**
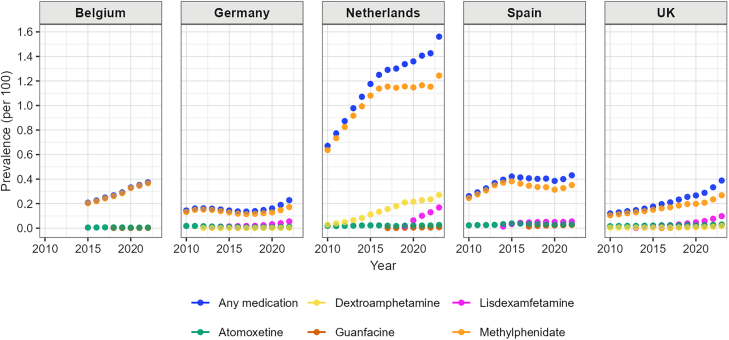
Yearly prevalence (per 100) of any ADHD medication, methylphenidate, atomoxetine, lisdexamfetamine, guanfacine, and dexamphetamine use, by country.

Over the study period, we observed that the overall prevalence of any ADHD medication use increased in all five countries, with the highest prevalence observed in the Netherlands (Fig. 2). The UK had the highest relative increase (223.9%) from 0.12% in 2010 to 0.39% in 2023, followed by the Netherlands (132.8% relative increase: from 0.67% in 2010 to 1.56% in 2023). In Belgium, the prevalence of ADHD medication use increased from 0.21% in 2015 to 0.37% in 2022. In Germany, the prevalence of ADHD medication use increased slightly from 0.14% in 2010 to 0.16% in 2012, then decreased to 0.14% in 2017. Since 2017, the prevalence increased substantially until the end of the study period, reaching 0.23% in 2022. In Spain, the prevalence increased from 0.26% in 2010 to 0.42% in 2015, then stabilised after 2015.

**Fig. 3:**
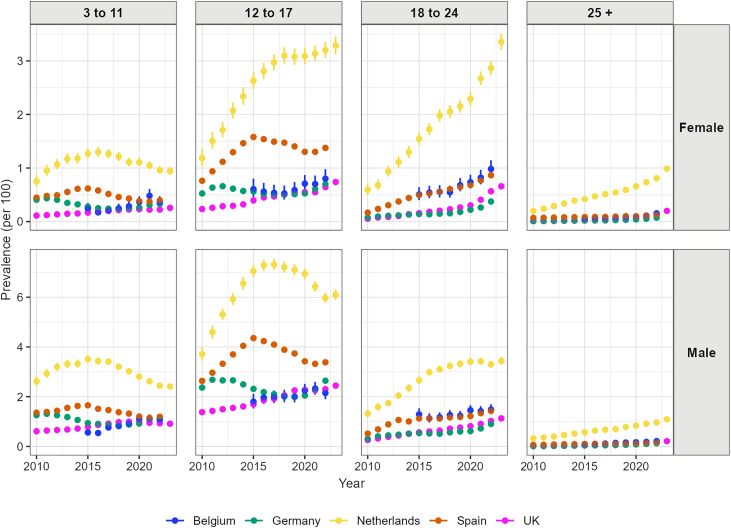
Yearly prevalence of any ADHD medication use, by age group, sex, and country.

We observed a shift in the age distribution of ADHD medication users, where young adults now surpass children in prevalence in some countries (Fig. 3, Appendix Figure S1). Among children aged 3 to 11, the prevalence of ADHD medication use increased in the UK (relative increase of 129.0% in females and 49.6% in males) and Belgium (relative increase of 47.5% in females and 91.7% in males) during the study period. In the Netherlands and Spain, the prevalence among children aged 3–11 years increased from 2010 to 2015, then decreased until 2023 in both males and females. Among children aged 12 to 17, we observed that prevalence increased in all five countries, especially among females. Prevalence among females increased from 1.18% to 3.29% in the Netherlands and from 0.23% to 0.74% in the UK during the study period. By the end of the study period, the prevalence among the 18 to 24 year-old group had overtaken the 3 to 11 year-old group in the UK, the Netherlands, and Spain for both males and females.

ADHD medication use increased dramatically among adults in all countries, especially adult females. In Belgium, prevalence among females aged 25 or older increased from 0.06% in 2015 to 0.15% in 2022. In Germany, the prevalence increased from 0.07% to 0.37% (relative increase 417.4%) among females aged 18 to 24, increased from 0.009% to 0.07% (relative increase 702.2%) among females aged over 25. In the Netherlands, prevalence increased from 0.60% to 3.35% (relative increase 462.4%) among females aged 18 to 24 and increased from 0.20% to 0.99% (relative increase 394.5%) among females aged over 25 years. In the UK, prevalence increased by 1770.1% (from 0.01% to 0.20%) in females and 1551.2% (from 0.01 to 0.21%) in males from 2010 to 2023 among those aged over 25. Among the 18 to 24 year-old group, prevalence increased by 1094.9% and 333.0% in females and males in the UK, respectively.

Methylphenidate was the most commonly used ADHD medication in all five countries, and the prevalence increased during the study period (Fig. 2, Appendix Figure S1). The trends over time of methylphenidate use were similar to the trends of any ADHD medication use. Following its introduction to the market, use of guanfacine and lisdexamfetamine increased steadily in all countries. By the end of the study period, lisdexamfetamine was the second most frequently used medication in Germany, Spain, and the UK. Use of dexamphetamine also increased in the Netherlands and the UK. Atomoxetine use was stable during the study period in Belgium. There was a slow decrease in atomoxetine use in Germany, from 0.018% in 2010 to 0.012% in 2022. Use of atomoxetine increased in the Netherlands, Spain, and the UK during the study period.

Appendix Figures S3–S7 present age- and sex-specific prevalence of individual ADHD medications. Across all five countries, prevalence rates for all five study medications increased among individuals aged 18 and older. Among children aged 3 to 17, the use of methylphenidate and atomoxetine decreased in most databases since 2015. Specifically, both medications decreased in Germany, the Netherlands, and Spain; atomoxetine use declined in the UK; and was very rarely recorded in Belgium. In contrast, the prevalence of lisdexamfetamine and guanfacine increased over the past decade in all databases, except Belgium, where no records of lisdexamfetamine were observed.

### Trends in incident use of ADHD medication

**Fig. 4:**
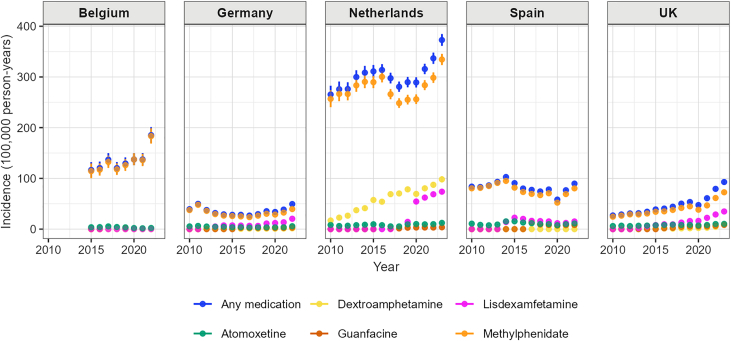
Yearly incidence of any ADHD medication, methylphenidate, atomoxetine, lisdexamfetamine, guanfacine, or dexamphetamine use, by country.

Fig. 4 shows the overall incidence rates of any ADHD medication use increased during the study period in Belgium (from 116.56 per 100,000 person-years (py) in 2015 to 185.26 in 2022), the Netherlands (from 265.44 per 100,000 py in 2010 to 372.86 per 100,000 py in 2023), and the UK (from 26.89 per 100,000 py in 2010 to 92.90 per 100,000 py in 2023). In Germany, the observed incidence rates of any ADHD medication use decreased from 39.30 per 100,000 py in 2011 to 26.72 per 100,000 py in 2017, then trended upwards until the end of the study period. In Spain, overall incidence rates increased from 2010 to 2014, then showed a downwards trend until 2018.

**Fig. 5:**
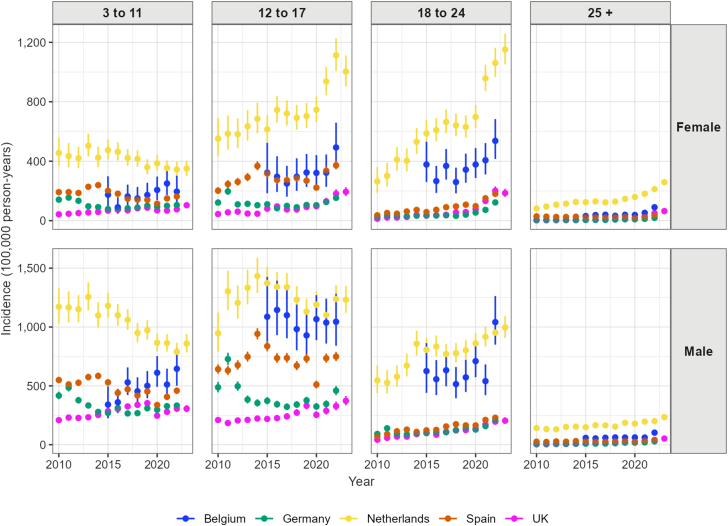
Yearly incidence of any ADHD medication use, by age-group, sex, and country.

The age-sex specific incidence rates of any ADHD medication use also differed between databases (Fig. 5, Appendix Figure S2). Among children aged 3 to 11, incidence rates of ADHD medication increased in both males and females in Belgium and the UK. In Germany, the Netherlands and Spain, we observed a decreasing trend in incidence rates among children aged 3 to 11 during the study period, especially among males. During the study period, incidence rates increased among females aged 12 to 17 in Belgium, the Netherlands and the UK. Among males, incidence rates showed a decreased trend since 2015 in Belgium, the Netherlands, and Spain until the pandemic. In the UK, incidence rates among males aged 12 to 17 increased during the study period. Among adults, the incidence rates of ADHD medication use increased in males and females in all five countries. For example, incidence rates among females aged 18 to 24 increased from 36.51 per 100,000 py in 2010 to 180.74 per 100,000 py in 2022, and from 13.23 per 100,000 py in 2010 to 185.52 per 100,000 py in 2023 in the UK.

While males continued to represent the majority of ADHD medication users, the male-to-female ratio has decreased in recent years (Appendix Figure S8) Among individuals under 18, the incidence of ADHD medication use remained substantially higher among males, particularly in the youngest age group. At the end of the study period, the male-to-female ratio was 3.29, 3.18, 2.45, 2.82, and 2.95 in Belgium, Germany, the Netherlands, Spain, and the UK, respectively.

Among adults, the discrepancy between males and females was less substantial. There have been more adult female than male initiators since 2021 in the Netherlands and since 2022 in the UK. In Germany, Belgium and Spain, while decreasing, the male-to-female ratio was still higher than 1. Among the 18–24 yrs old group, the male-to-female ratio ranged from 1.53 in Germany to 0.81 in the UK at the end of study period. Among people aged over 25, the male-to-female ratio changed from 1.87 to 1.14 in Belgium, from 1.4 to 1.53 in Germany, from 1.74 to 0.9 in the Netherlands, from 0.9 to 1.18 in Spain, and from 1.26 to 0.81 in the UK.

Changes in incidence rates of individual ADHD medications differed by database (Appendix Figures S9–S13). During the study period, we observed that overall incidence rates of lisdexamfetamine and dextroamphetamine use increased in the UK, the Netherlands, and Germany. The incidence rates of lisdexamfetamine use decreased in Spain from 2015 to 2020. Incidence rates for all medications were lower in 2020, reflecting a reduction in healthcare utilisation during the pandemic.

### Treatment adherence

Table 2 summarises the PPC among people who initiated any ADHD medication during the first 5 years after initiation. After one year of medication initiation, among those who remained in follow-up, 14.9%, 16.0%, 43.9%, and 30.8% of initiators in Germany, the Netherlands, Spain, and the UK, respectively, have ongoing ADHD medication coverage based on prescription records. As expected, the PPC dropped at 1 and 2 years after initiation (for example, from 28.7% to 14.9% and then 8.4% in Germany). We observed a higher PPC in Spain during the follow-up compared to other databases. In the stratified analysis, higher PPC was observed among children aged 3–11 years than in other age groups, with the next highest PPC observed in individuals aged over 25. Within each age group, the PPC between males and females was similar (Appendix Figure S14).

**Table 2:** Proportion of patients covered by ADHD medication after initiation during 5-years follow-up.

	Germany	Netherlands	Spain	UK
Time				
180 days	28.7% [28.3%–29.2%]	29.4% [29.0%–29.9%]	62.6% [62.2%–63.0%]	44.1% [43.5%–44.7%]
1 year	14.9% [14.5%–15.2%]	16.0% [15.7%–16.4%]	43.9% [43.5%–44.3%]	30.8% [30.2%–31.4%]
2 years	8.4% [8.1%–8.7%]	9.9% [9.6%–10.2%]	29.8% [29.4%–30.2%]	21.6% [21.1%–22.2%]
3 years	6.0% [5.7%–6.4%]	7.1% [6.8%–7.4%]	23.2% [22.8%–23.6%]	17.3% [16.7%–17.9%]
4 years	4.9% [4.5%–5.2%]	5.5% [5.2%–5.8%]	18.7% [18.3%–19.1%]	13.9% [13.3%–14.6%]
5 years	4.0% [3.6%–4.3%]	4.5% [4.2%–4.8%]	15.5% [15.1%–15.8%]	12.2% [11.5%–12.8%]

In the sensitivity analysis where we applied a 90-day grace period, higher PPC was observed. After one year of initiation 36.2%, 33.0%, 53.5%, and 56.9% of participants have ongoing ADHD medication coverage in Germany, the Netherlands, Spain, and the UK respectively (Appendix Table S1).

## Discussion

In this population-based, multinational study with five European databases from the DARWIN-EU® network, we observed a substantial increase in ADHD medication use since 2010. Use of guanfacine and lisdexamfetamine increased in all countries following market approval. While the extent and trend of use over time among children and adolescents varied across countries, we observed that both the prevalence and incidence of ADHD medication use increased among adults in all countries, with the disparities between males and females decreasing over year. Adherence to medication varied across countries but was overall low after initiation. Psychiatric comorbidities and concomitant medication use were frequent among people who initiated ADHD medication, especially among adults.

Trends in ADHD medication use among both children and adults have been studied previously with different databases across the world.^5^, ^6^, ^7^^,^^11^^,^^16^, ^17^, ^18^, ^19^, ^20^, ^21^, ^22^, ^23^ However, previous studies usually used older data, especially before the Covid-19 pandemic, focused on children rather than adults, included only single database, and most of them estimated only prevalence use as there was no reliable denominator estimate. There were five previous multinational studies that compared prevalences of ADHD medication use, including two cross-continental studies with data from Europe,^6^^,^^19^ and three from Nordic countries.^7^^,^^21^^,^^24^ Following previous studies on trends of ADHD medication use,^5^, ^6^, ^7^^,^^11^^,^^16^, ^17^, ^18^, ^19^, ^20^, ^21^, ^22^, ^23^ our study documented a continued overall increase in use of ADHD medication in Belgium, Germany, the Netherlands, and the UK. Guanfacine and lisdexamfetamine received approval for ADHD treatment during the study period, leading to increased use after their market approval. The increased prevalence of overall ADHD medication use as well as individual medication align with analyses based on pharmaceutical sales data.^5^

We observed that trends in medication use among children and adolescents varied across countries, likely reflecting differences in clinical guidelines, prescribing cultures, regulatory policies, and the availability or preference for non-pharmacological interventions.^25^, ^26^, ^27^ For example, in the Netherlands, although overall ADHD medication use increased during the study period, we observed a decline in prevalence after 2015 among males aged 3–17 and females aged 3–11, with a slower rate of increase among adolescent females. This trend may be partly explained by the Youth Act implemented by the Dutch government, which aimed to reduce medication use in mental healthcare among children and adolescents.^28^

While direct comparison of estimated prevalence is not possible due to differences in data source, study period, and study population definition and stratification, the country-specific and between country trend in the current study is comparable to published literatures. For example, with data from five countries 2005 to 2012, Bachmann et al. reported that among children and adolescents, the prevalence of ADHD medication use was higher in the US, followed by the Netherlands, Germany, Denmark, then the UK.^19^ In the current study among the 3–17 years group, we observed highest prevalence of use in the Netherlands, followed by Spain, Germany, and then the UK. Another study from Germany reported that prevalence among children decreased from 2012 to 2018. Our results showed similar trend, and further observed an increased in prevalence from 2018 to 2022.^20^ Studies using electronic health records from the UK showed that the prevalence use of ADHD medication increased in both males and females across all age groups during 1995–2018.^16^, ^17^, ^18^ In our study, we observed that the increase trends continued until 2023. The observed increased trend is align with the published statistics on medicines used in mental health England, which reported the number of stimulant use in England 2015 to 2024.^29^ For countries where publicly available figures on medication use were available, estimates from the current study are in line with those statistics.^30^

Changes in prevalences of medication use can be explained by both changing in number of incident users and changing in treatment duration once ADHD medication is initiated, as well as changes of alternative medications. However, fewer published studies have estimated the incidence use of ADHD medications.^16^^,^^18^^,^^22^ A previous study using the UK IQVIA Medical Research Data estimated the incident use of any ADHD medication from 2000 to 2018, and reported that the incidence increased in all age groups during the study period.^18^ In this study, we observed that in the UK data, the incident use of all five ADHD medication increased during the study period, with a drop in 2020 due to the reduced health care access during the COVID-19 pandemic.

We observed dramatic rise in ADHD medication use among adults, especially among females. Among children and adolescent, while decreasing over year, the male-to-female ratio remained higher than 2 in most countries at the end of study period. Among adults aged over 18, there was 2–15 folds increase in prevalence of use during the study period, with the most pronounced increases observed in females, especially in the UK. In the Netherlands and the UK, incidence use among adults surpassed males by the end of the study period. The increase in both prevalence and incidence use among adults reflects the increased awareness of adult ADHD, especially among females.^31^ Since 2013, the DSM-IV updated the diagnostic criteria for ADHD, raising the age of symptom onset to before 12 years from before 7, and decreasing the minimum symptom requirement to five for adults and adolescents aged 17 and older.^32^ Other factors include the increased online interest in ADHD related topic in recent years, impact of the COVID-19 pandemic,^33^ and the discussion on modification of the diagnostics criteria of requiring early onset symptoms.

The current study used PPC to estimate medication adherence, which measures the proportion of live patients currently covered by treatment and allows treatment break. A recent study across eight countries reported that 39–65% of participants remained on treatment one year after initiation, with adherence highest among children.^11^ While we observed similar patterns across age groups, the estimated PPC from our study were lower. This may be due to the different definition of discontinuation. In our study, we defined treatment episode using a 30-day gap, while Brikell et al. used two dispensations within 180 days, translating to a 150-day period, assuming one-month prescription. In the sensitivity analysis using an extended 90-day grace period, our adherence estimates aligned with those reported by Brikell et al. While the PPC method appeared less sensitive to grace period definitions, further research is needed to assess their impact and inform best practices for defining grace periods.

In our study, we observed that while the prevalence and incidence rates of ADHD medication use in the Netherlands were higher than in other databases, adherence to ADHD medication was lower than other countries. Conversely, the prevalence and incidence rates of ADHD medication use were lower in the UK and Spain, but we observed higher PPC in these two countries. This could reflect different clinical practices in each country, where pharmacological treatment may be prioritised for people who are more likely to benefit from and adhere to m treatment, or alternatively, offered to a broader population.^16^^,^^19^

We described the characteristics of individuals at the time of ADHD medication initiation, and showed many participants had psychiatric or mental health conditions and previously used psycholeptic medications, especially among adults. However, in clinical trials of ADHD medications, people with these conditions or medication use were usually excluded from participation.^34^ Future research is warranted to assess the real-world utilisation, safety, and effectiveness of ADHD medication among this population. It is also important to note that characteristics of study population should be interpreted within the context of each country or database. Clinical practices vary across countries in terms of prescribing psychotropics and ADHD medications, contributing to the observed differences in results. Therefore, we suggest that information on prior psychotropic use be used for within-country or within-database comparisons between demographic groups, rather than for cross-country comparisons.

Our findings on trends in ADHD medication use should be interpreted in the context of the underlying condition. Although these trends varied across countries, the prevalence of medication use remains substantially lower than the estimated prevalence of ADHD. Globally, ADHD affects approximately 8% of children and adolescents and 3% of adults.^1^^,^^2^ While it is true that not all individuals with ADHD require medication, our findings suggest that a substantial proportion may not be receiving pharmacological treatment. The high rates of comorbidity and co-medication among individuals received ADHD medication suggest that those with less complex clinical profiles may be less likely to receive pharmacological treatment.

This study has several key strengths. We included longitudinal data covering periods up to and beyond the COVID-19 pandemic, offering an up-to-date perspective on ADHD medication use within the DARWIN EU® network. Unlike many previous studies that were typically limited to a single database, focused primarily on children, or estimated only medication prevalence due to the lack of reliable denominator data, our study leveraged multiple, population-based, routinely collected healthcare databases from different countries. This enabled robust estimation of both the incidence of ADHD medication use and detailed characterisation of medication users across diverse healthcare settings. The use of the OMOP CDM and standardised analytics allowed for federated execution across all participating databases, enhancing the consistency, reliability, and scalability of our findings. Furthermore, the full availability of our analytical code ensures transparency and reproducibility, supporting open science and enabling future research.

The results of this study must be taken in the context of the following limitations. First, the study used routinely collected health care data that were not collected for research purpose. Therefore, a record of a prescription does not mean that the individual actually took the drug. Second, variation in the proportion of ADHD medication initiators with a recorded diagnosis of ADHD and certain mental health conditions likely reflects differences in how and where these conditions are diagnosed and recorded. As a result, information on comorbidities may be incomplete. In databases like the UK CPRD, which primarily contain general practice records, ADHD diagnoses may be under-recorded. In contrast, databases from Belgium and Germany capture a broader range of healthcare settings including specialists, resulting in more complete diagnostic information. Caution is therefore warranted when interpreting cohort characteristics across databases. As this was a descriptive analysis, the male-to-female ratio was estimated as a crude measure, without adjustment for factors such as differential care-seeking behaviour. These results should be interpreted with caution. The current study did not include information on ethnicity or socioeconomic status, which are important factors for ADHD medication prescribing and use. However, these variables were not consistently available across participating databases.^35^^,^^36^ Future research should consider stratified analyses by ethnicity and socioeconomic group to better understand health inequalities in medication use, especially in European context.

In conclusion, this population-based, multinational study provided updated trends of ADHD medication use among both children and adults in five European countries. Our findings provide useful information into patterns of medication use and can inform resource allocation in healthcare setting to support proactive planning and mitigate potential shortages, especially for countries included in the study. Future study should focus on assess the safety and effectiveness of ADHD medication, particularly among adults and among people with psychiatric comorbidities or comedications.

## Contributors

XL, YG, and EB had full access to the aggregate analysis data in the study and take responsibility for the integrity of the data and the accuracy of the data analysis. XL and DAP were responsible for funding acquisition, YG, EB, and XL were responsible for the development of code for statistical analyses. AGP, APC, WYM, IK, and MM were responsible for study code execution in the respective databases. YG, EB, and XL were responsible for interpretation and visualisation of the results. XL drafted the manuscript. All authors contributed to the conceptualisation of the study and writing, reviewing, and editing of the manuscript. All authors confirm that they had full access to all the data in the study and accept responsibility to submit the article for publication.

*Database specific:* CPRD GOLD data mapping: WYM and AD. Access and verification to CPRD GOLD data: AD. Access and verification to IPCI data: MM and KV. Access and verification to SIDIAP data: AGP, APC and TDS. Access to IQVIA LPD Belgium and IQVIA DA Germany: IK, JB.

## Data sharing statement

Country-specific regulations and laws prohibit sharing or making the individual-level data in this study publicly available. Access to these data is not possible without the permission of the relevant approving human research ethics committees or the data custodians.

## Declaration of interests

The manuscript received support from the European Medicines Agency through the DARWIN Coordination Centre. Data partners’ role was only to execute code at their data source. These people do not have an investigator role. All other authors declare no conflicts of interest related to this study.

The authors report the following conflicts of interest not related to this study: DPA's research group from the University of Oxford has received research grants from the European Medicines Agency, from the Innovative Medicines Initiative, from Gilead Science, from Theramex, and from UCB Biopharma. Janssen has funded or supported training programmes organised by DPA's department. DPA sits in the Board of the EHDEN Foundation. KV works for a research department who in the last 3 years received unconditional research grants from UCB and Johnson and Johnson. MM works for a research group that in the past 3 years receives/received unconditional research grants from Chiesi, UCB, Amgen, Johnson & Johnson, Innovative Medicines Initiative, and the European Medicines Agency. TDS works for a research group that in the past 3 years receives/received unconditional research grants from UCB, Johnson & Johnson, Innovative Medicines Initiative, and the European Medicines Agency.
