# Supplementary material for: Trends in use of Attention-Deficit Hyperactivity Disorder medications among children and adults in five European countries, 2010 to 2023: a population-based observational study
Source: Lancet Reg Health Eur. 2026 Jan 21;61:101556. doi: 10.1016/j.lanepe.2025.101556 (PMC12882648; doi:10.1016/j.lanepe.2025.101556)
Supplement: Supplementary Figures and Table [file mmc1.docx]

**Supplementary materials for: Trends in use of Attention-Deficit Hyperactivity Disorder medications among children and adults in five European countries, 2010 to 2023: a population-based observational study**

## **Appendix 1. Description of study databases**

**IQVIA Longitudinal Patient Data Belgium [IQVIA LPD Belgium]**

IQVIA Longitudinal Patient Data (LPD) Belgium is a database of pseudonymised electronic medical records from general practices (GPs) in Belgium since 2005.(“IQVIA Longitudinal Patient Data - Belgium” n.d.) The database encapsulates records of approximately 10% of Belgian patient population.

This patient-level database captures patient demographics, diagnoses (using a specific diagnostic coding system that can be bridged with ICD-10-CM codes). In addition, it encompasses medical history, prescription data (associated with a hard-coded diagnosis), as well as supplementary metrics such as anthropometric measures (height, weight), vital signs (blood pressure) and results from laboratory tests. All patients in the database are pseudonymised and can be followed longitudinally based on a unique identifier. Strict attention to confidentiality is present at every stage of data collection, storage and analysis in accordance with General Data Protection Regulation (GDPR)and Belgian Ethics Committees recommendations. IQVIA LPD Belgium database is nationally representative in terms of both geographical coverage and patient demographic characteristics, including age and sex.

- 1. **IQVIA Disease Analyzer Germany [IQVIA DA]**

IQVIA Disease Analyzer (DA) Germany is a database of de-identified electronic medical records from specialised and general primary practices in Germany since 1992.(“IQVIA Disease Analyzer Germany” n.d.) This dataset encompasses approximately 3% of all outpatient practices within Germany, ensuring a substantial representation of the national healthcare landscape. The sampling methods used for practice selection, taking into account physician’s demographics, specialty focus, community size category and federal state location, was instrumental in constructing a database that accurately mirrors the diverse spectrum of healthcare providers in the country. Consequently, data within IQVIA DA Germany database has been demonstrated to be representative of general and specialised practices throughout Germany.

The database contains demographics records, basic medical data, disease diagnosis according to International Classification of Diseases, 10th revision (ICD-10), and prescription records. While the database partly records information on deaths and procedures, it currently does not support linkage with external data sources. Routine updates are conducted at regular intervals. The quality of data is assessed based on several criteria including completeness of information and correctness (e.g. linkage between diagnosis and prescriptions).

- 1. **Integrated Primary Care Information Project [IPCI], The Netherlands**

The Integrated Primary Care Information (IPCI) database is a longitudinal observational database containing routinely collected data extracted from computer-based patient records of a selected group of general practitioners across the Netherlands.(“Integrated Primary Care Information (IPCI)” n.d.) IPCI was started in 1992 by the department of Medical Informatics of the Erasmus University Medical Center in Rotterdam. The current database includes patient records from 2006 onwards, when the size of the database started to increase significantly. The demographic composition of the IPCI population mirrors that of the general Dutch population in terms of age and sex. Although the geographical spread is limited, GP practices are located in urban and non-urban areas.

Patient-level data includes demographic information, patient’s complaints and symptoms, diagnoses, laboratory test results, lifestyle factors and correspondence with secondary care, such as referral and discharge letters.

- 1. **Sistema d’Informació per al Desenvolupament de la Investigació en Atenció Primària [SIDIAP], Spain**

The Information System for Research in Primary Care (SIDIAP) is a dynamic database of pseudo-anonymised electronic health records of the primary care patient population in Catalonia, Spain.(“The Information System for Research in Primary Care (SIDIAP)” n.d.) It contains data of approximately 80% of the Catalan population registered in over 280 primary care practices throughout Catalonia since 2005.

The database contains data recorded in primary care centres on a daily basis. Additionally, it integrates data from external sources including biomarkers data from laboratories and records of drug prescription and dispensation. The dataset covers demographics, all-cause mortality, disease diagnoses classified under ICD-10, prescription and dispensation records of drugs, results of laboratory tests, socio-economic indicators, vaccination records, lifestyle information, parent–child linkage and various clinical parameters. Additional data from other data sources such as hospital discharges, mental health centres or specific disease registries can be obtained through diverse linkages. The demographic composition within SIDIAP closely mirrors that of the broader Catalan population, encompassing a representative spectrum of geographic distribution, age, and sex proportions. The database is updated every 6 months.

- 1. **Clinical Practice Research Datalink [CPRD], the UK**(Sanchez-Santos et al. 2025)

The Clinical Practice Research Datalink (CPRD) GOLD is a database of anonymised electronic health records (EHR) from General Practitioner (GP) clinics in the UK that use the Vision® software system for their management. The source population encompasses 98% of the UK, registered with GPs responsible for non-emergency care and referrals. Participating GPs provide CPRD EHR for all registered patients who did not specifically request to opt out of data sharing. Covering 4.6% of the current UK population, GOLD includes 4.9% of contributing GP practices, providing comprehensive information within its defined source population.(“Clinical Practice Research Datalink (CPRD) GOLD” n.d.) GOLD contains data from all four UK constituent countries and the current regional distribution of its GP practices (among the 4.9% in the UK) is 5.7% in England, 55.6% in Scotland, 28.4% in Wales, and 10.2% in Northern Ireland (May 2022). GOLD data include patient’s demographic, biological measurements, clinical symptoms and diagnoses, referrals to specialist/hospital and their outcome, laboratory tests/results, and prescribed medications.

**Reference**

“Clinical Practice Research Datalink (CPRD) GOLD.” n.d. Accessed December 5, 2024. https://catalogues.ema.europa.eu/node/1026/administrative-details.

“Integrated Primary Care Information (IPCI).” n.d. Accessed December 5, 2024. https://catalogues.ema.europa.eu/data-source/42618.

“IQVIA Disease Analyzer Germany.” n.d. Accessed December 5, 2024. https://catalogues.ema.europa.eu/node/1021/administrative-details.

“IQVIA Longitudinal Patient Data - Belgium.” n.d. Accessed December 5, 2024. https://catalogues.ema.europa.eu/node/951/administrative-details.

Sanchez-Santos, Maria T., Eleanor L. Axson, Daniel Dedman, and Antonella Delmestri. 2025. “Data Resource Profile Update: CPRD GOLD.” *International Journal of Epidemiology* 54 (4). https://doi.org/10.1093/ije/dyaf077.

“The Information System for Research in Primary Care (SIDIAP).” n.d. Accessed December 5, 2024. https://catalogues.ema.europa.eu/node/1019/administrative-details.

**Figure S1. Annual prevalence and incidence of methylphenidate, atomoxetine, lisdexamfetamine, guanfacine, or dexamphetamine use (note: variation in y-axes)**


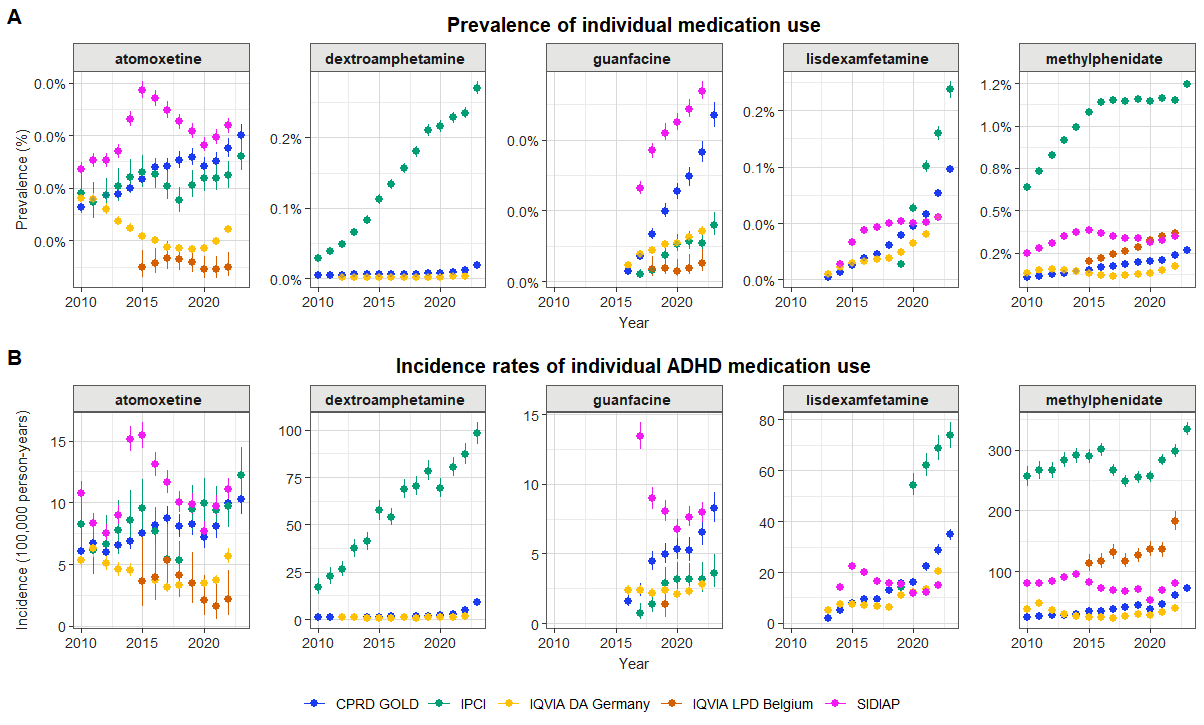


**Figure S2. Prevalence and incidence rates of any ADHD medication use, by age group and country.**


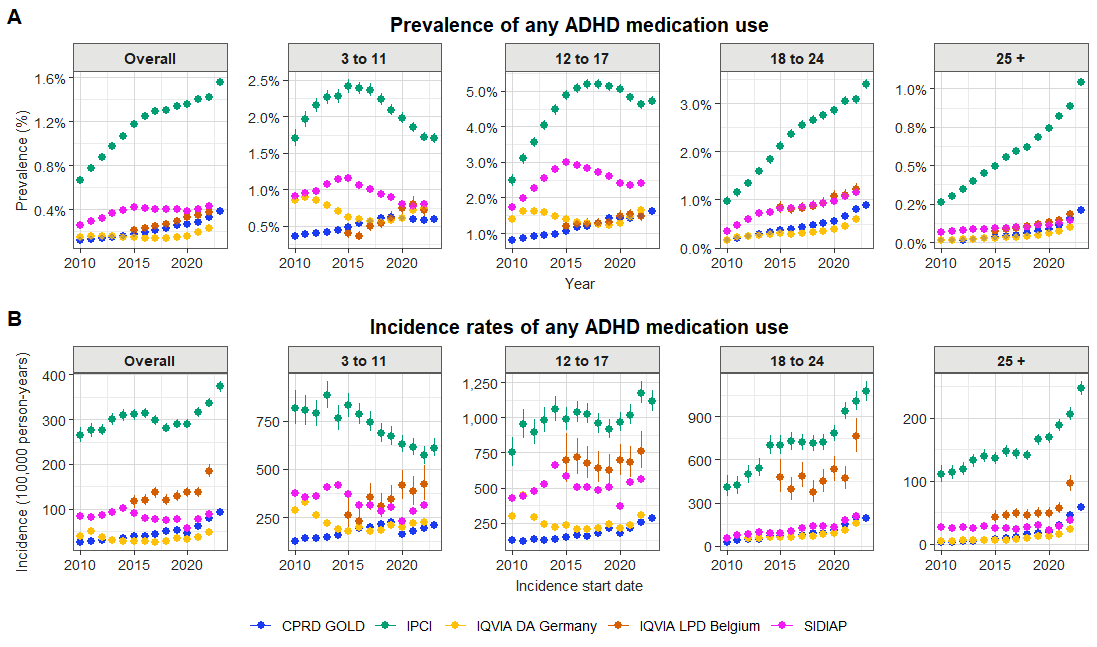


**Figure S3. Prevalence use of ADHD medication by age group and sex: IQVIA LPD Belgium.**


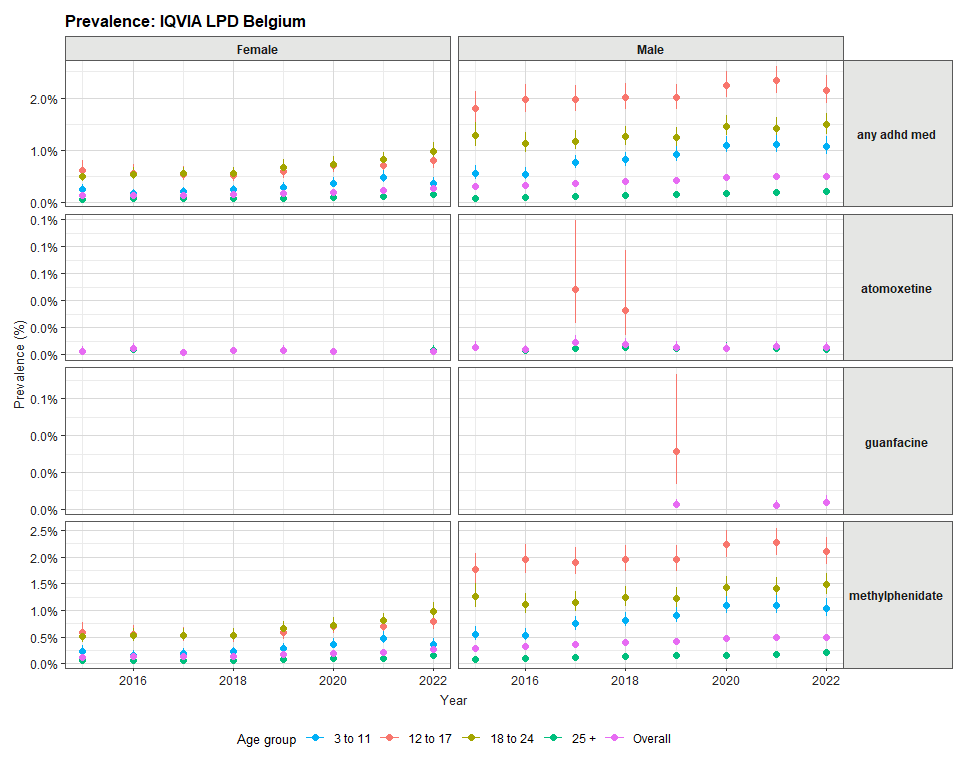


**Figure S4. Prevalence use of ADHD medication by age group and sex: IQVIA DA Germany.**


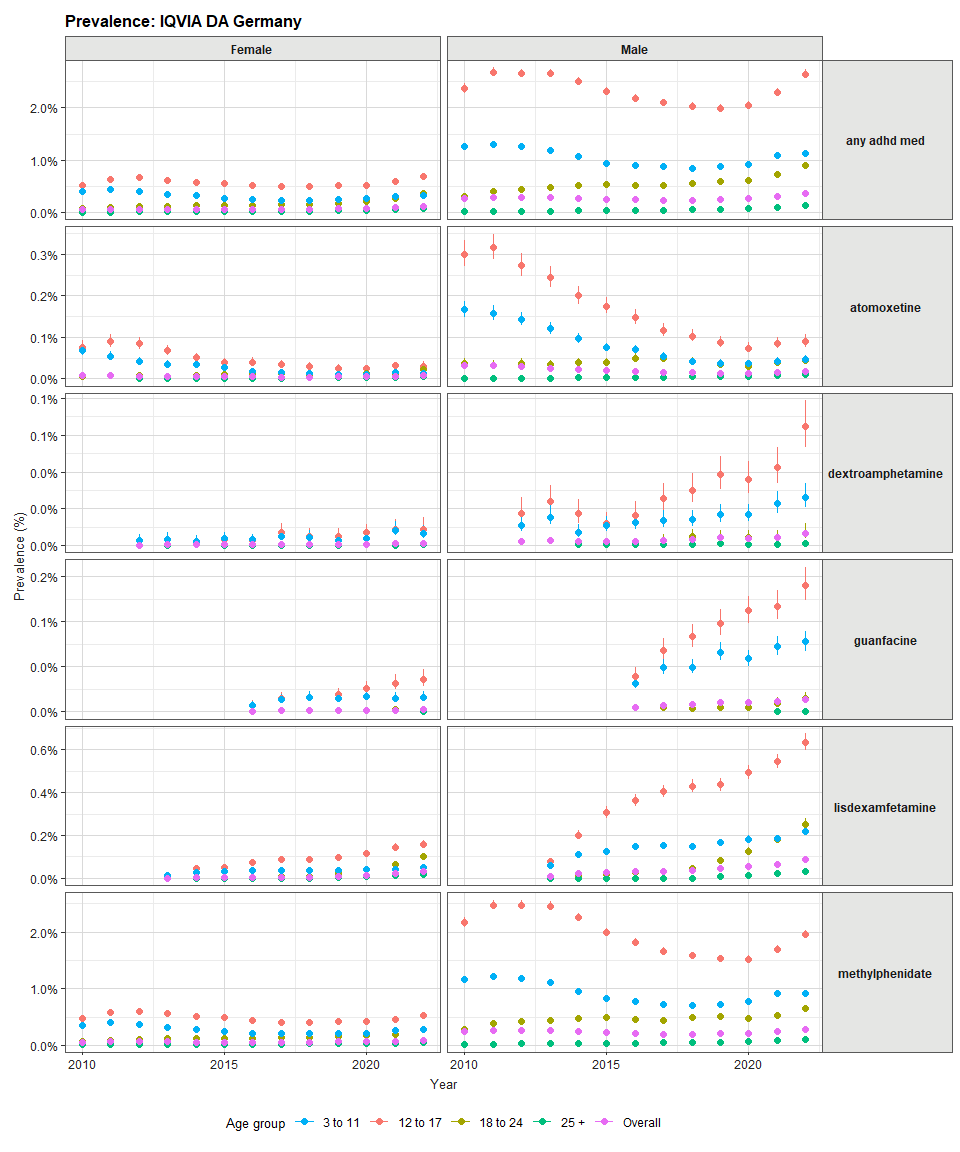


**Figure S5. Prevalence use of ADHD medication by age group and sex: IPCI.**


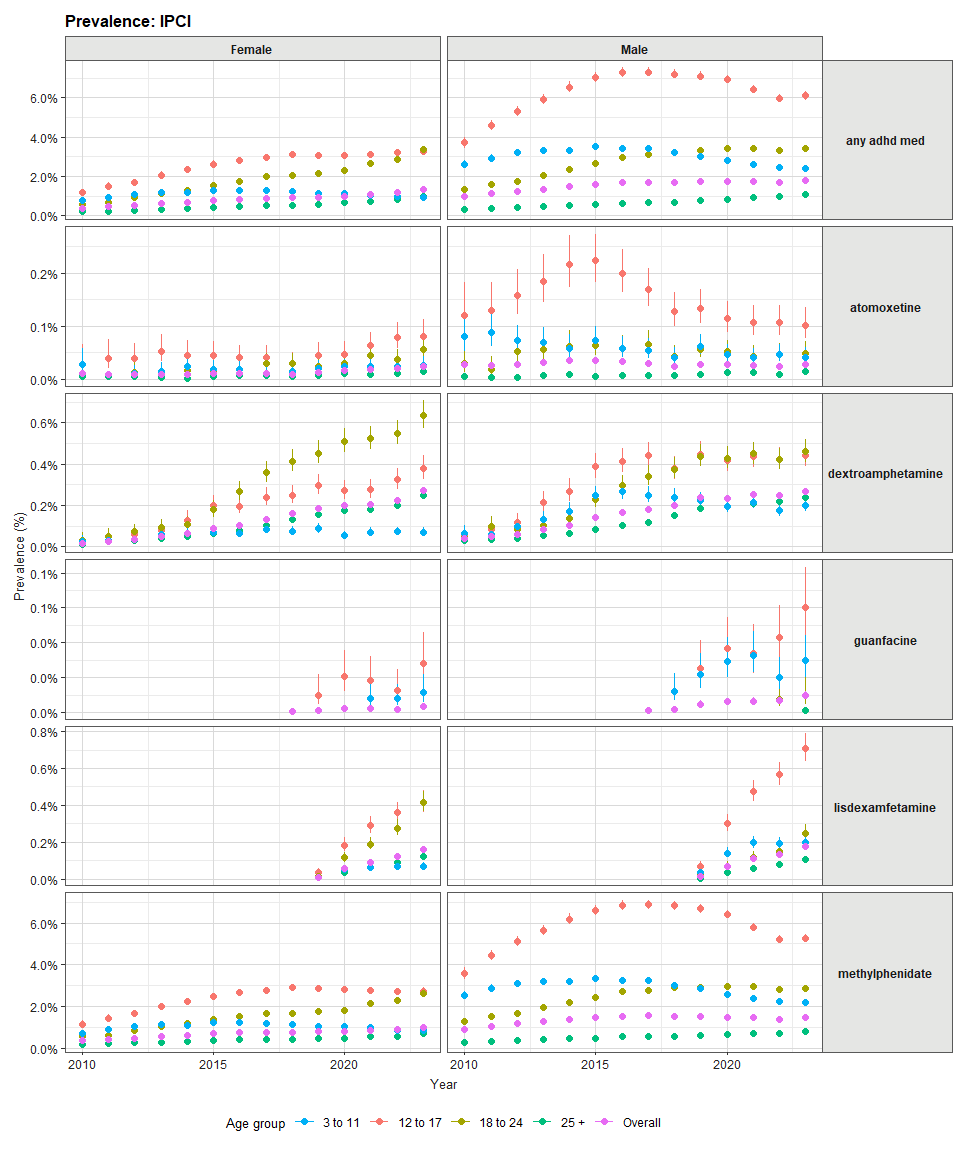


**Figure S6. Prevalence use of ADHD medication by age group and sex: SIDIAP.**


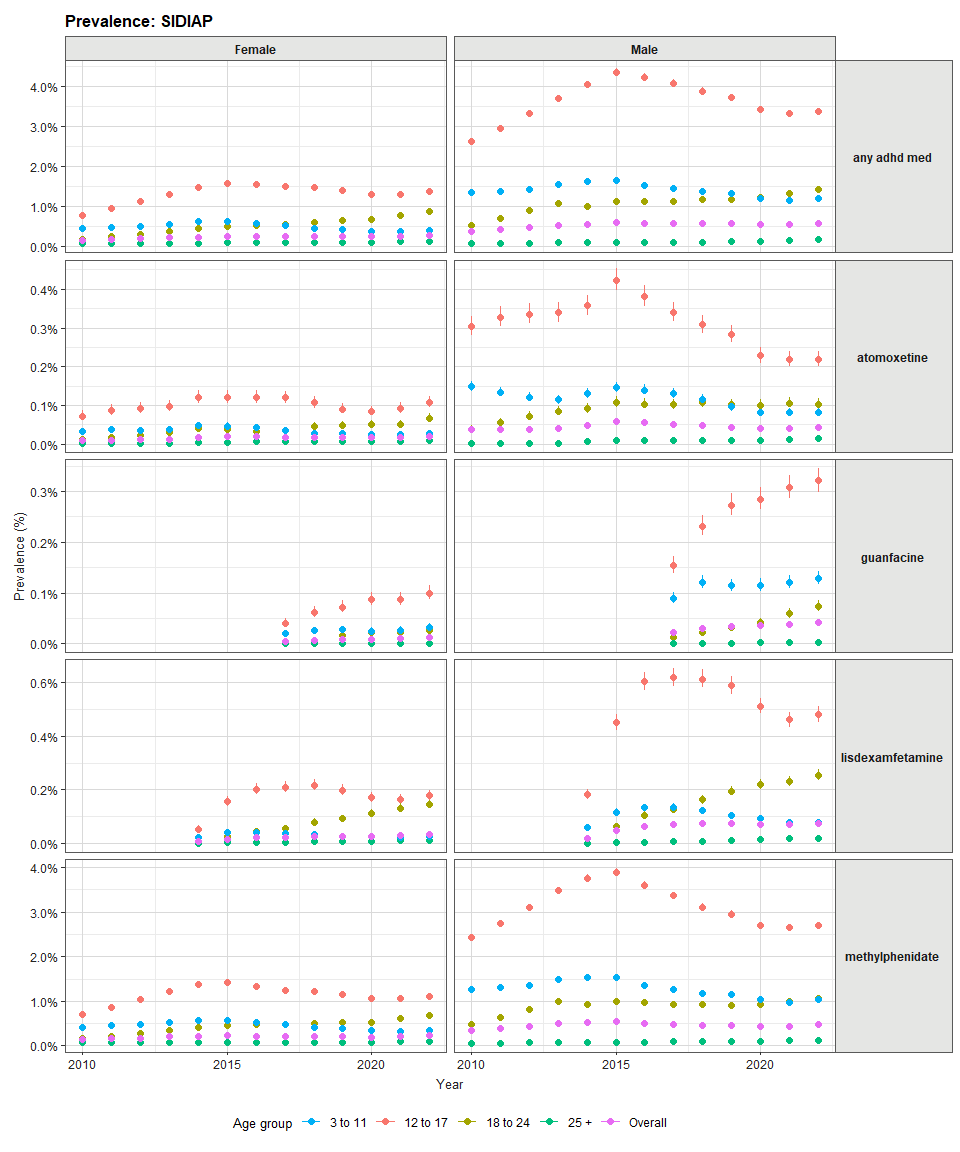


**Figure S7. Prevalence use of ADHD medication by age group and sex: CPRD GOLD.**


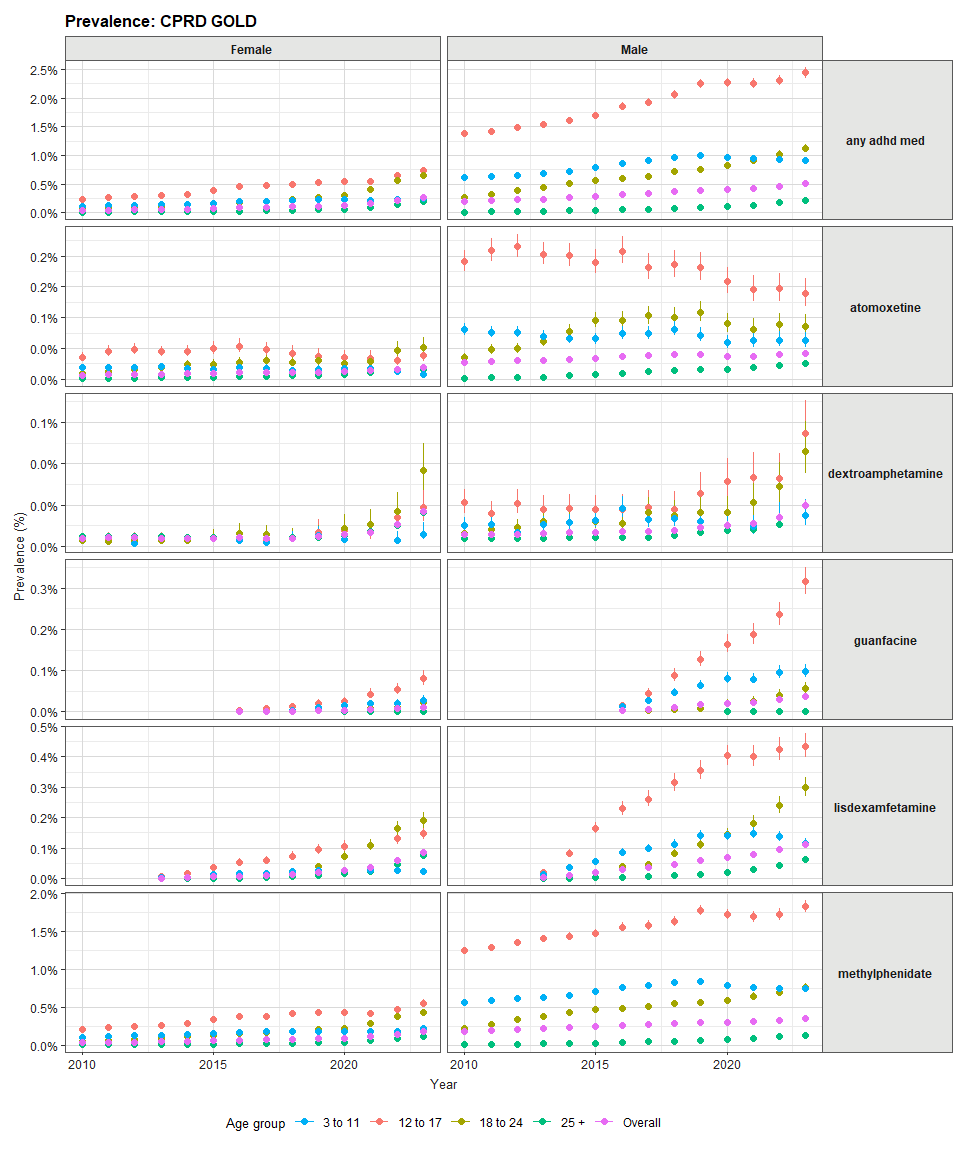


**Figure S8. Male-to-female incidence rate ratio of any ADHD medication use by age group 2010 TO 2023. A male/female ratio >1 indicates higher use among males.**


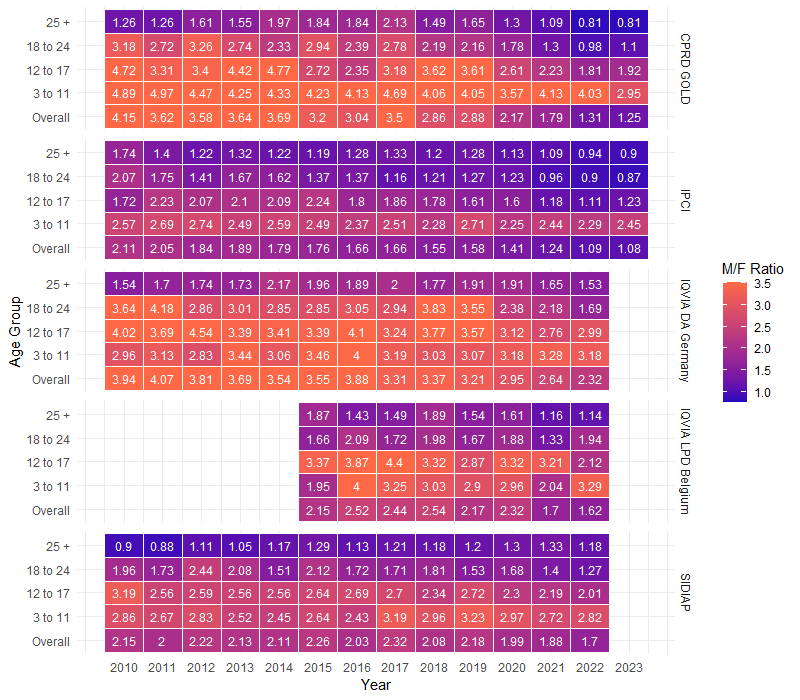


**Figure S9. Incidence rates of ADHD medication by age group and sex: IQVIA LPD Belgium.**


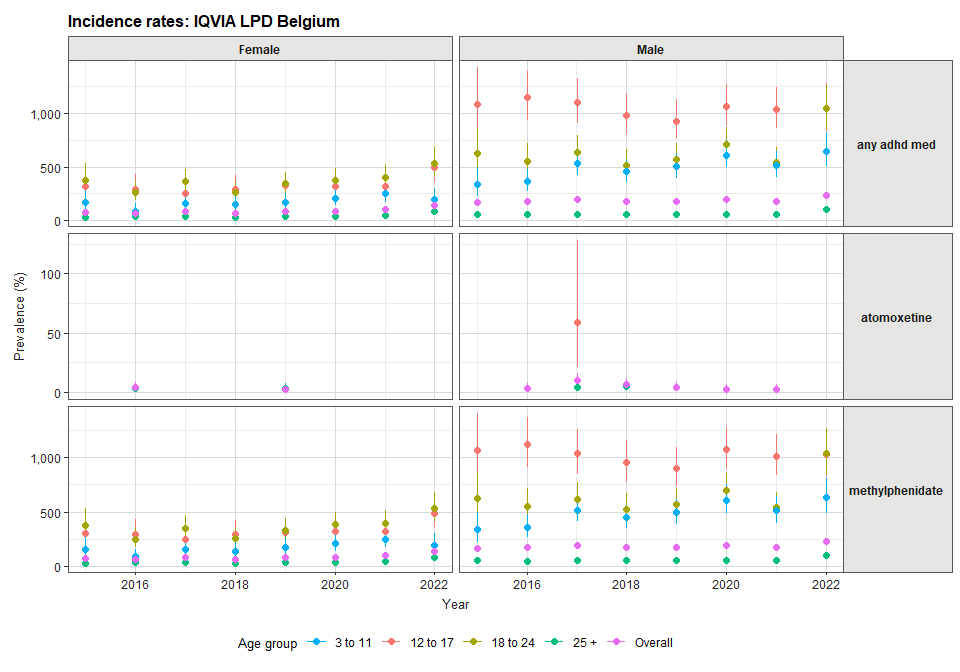


**Figure S10. Incidence rates of ADHD medication by age group and sex: IQVIA DA Germany.**


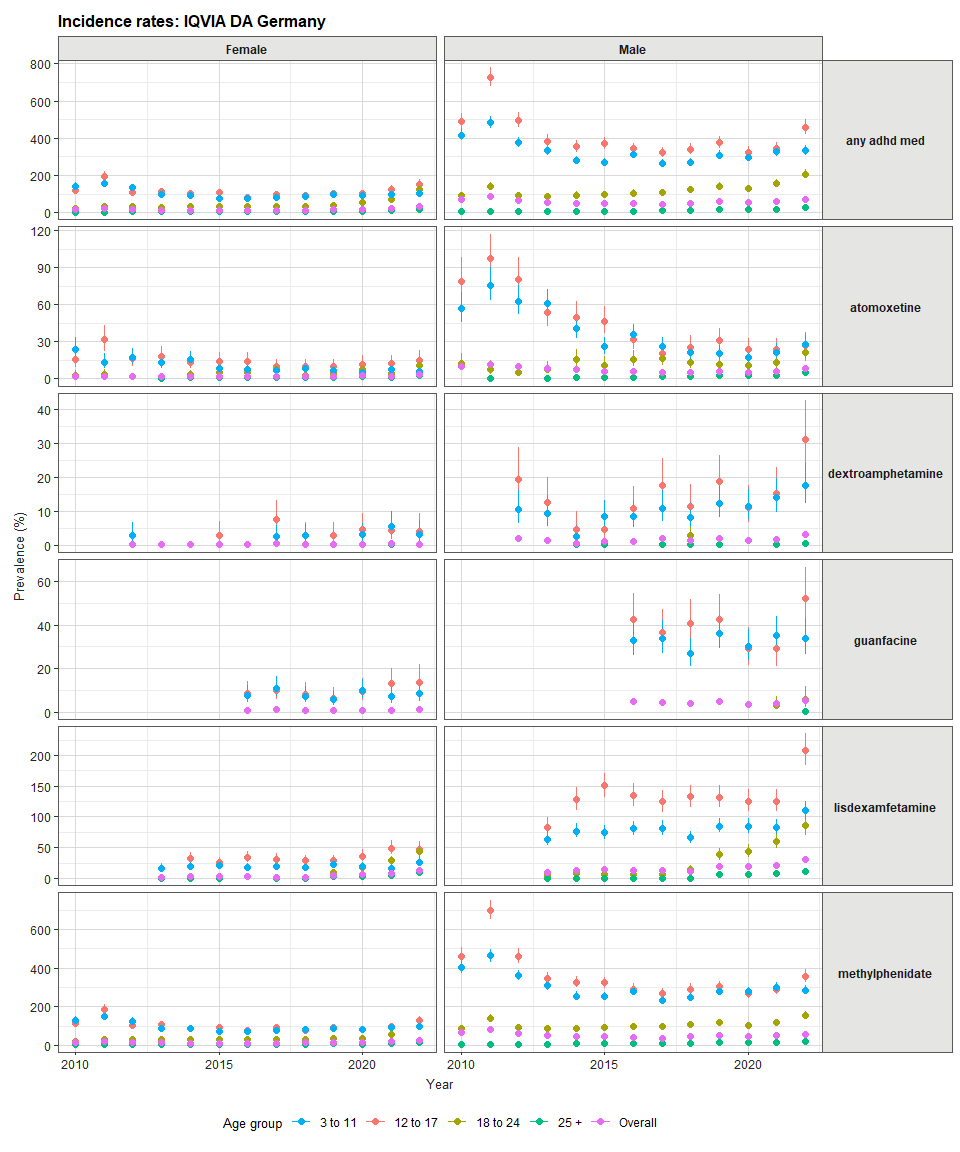


**Figure S11. Incidence rates of ADHD medication by age group and sex: IPCI.**


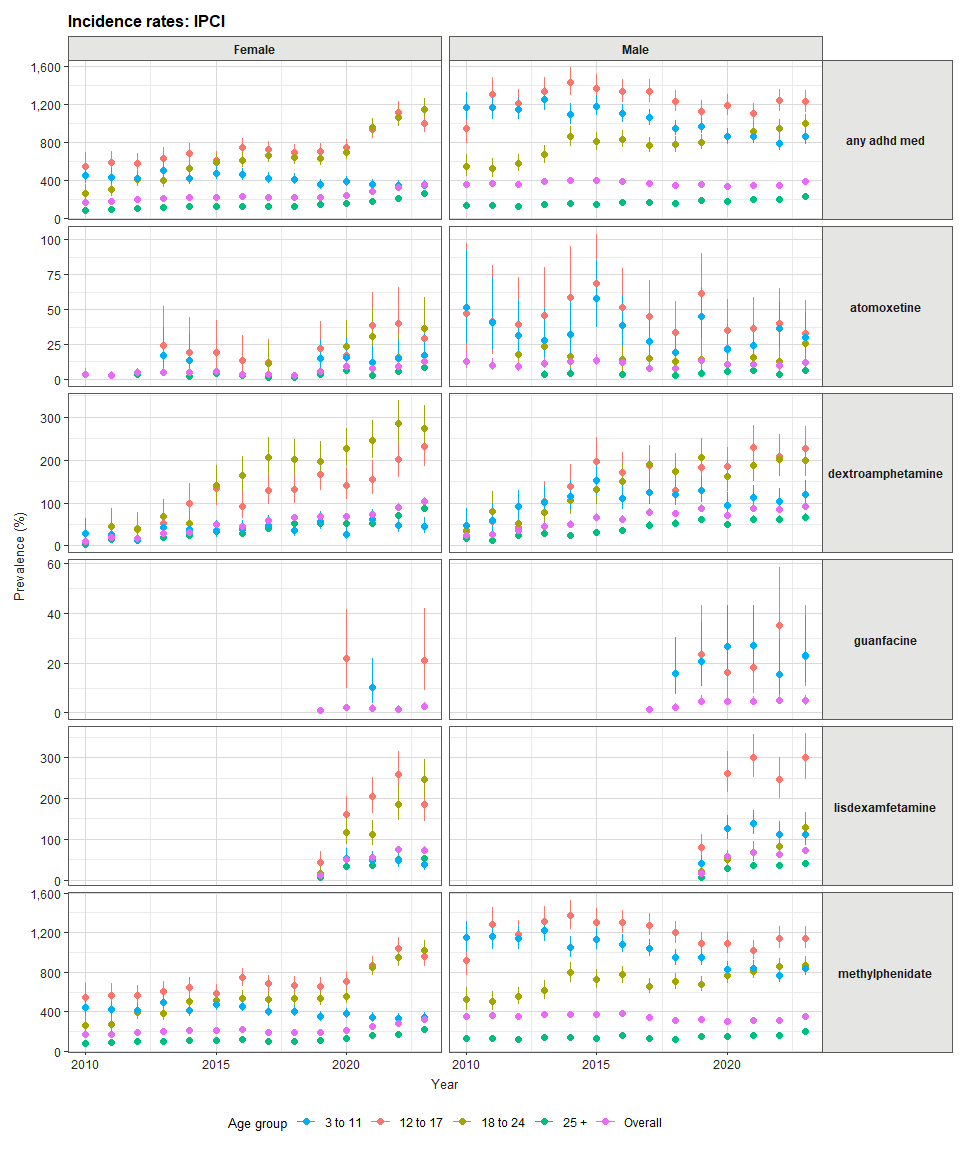


**Figure S12. Incidence rates of ADHD medication by age group and sex: SIDIAP.**


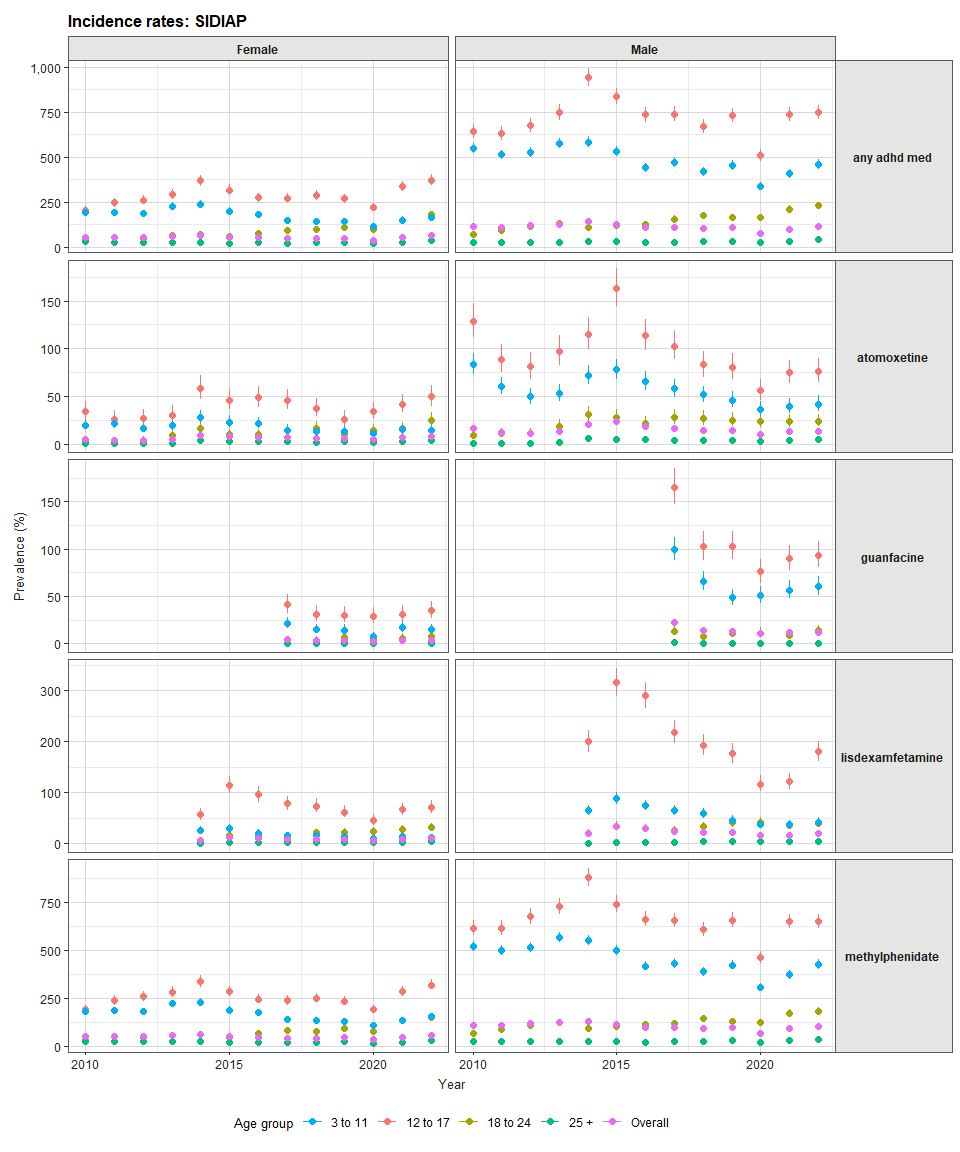


**Figure S13. Incidence rates of ADHD medication by age group and sex: CPRD GOLD.**


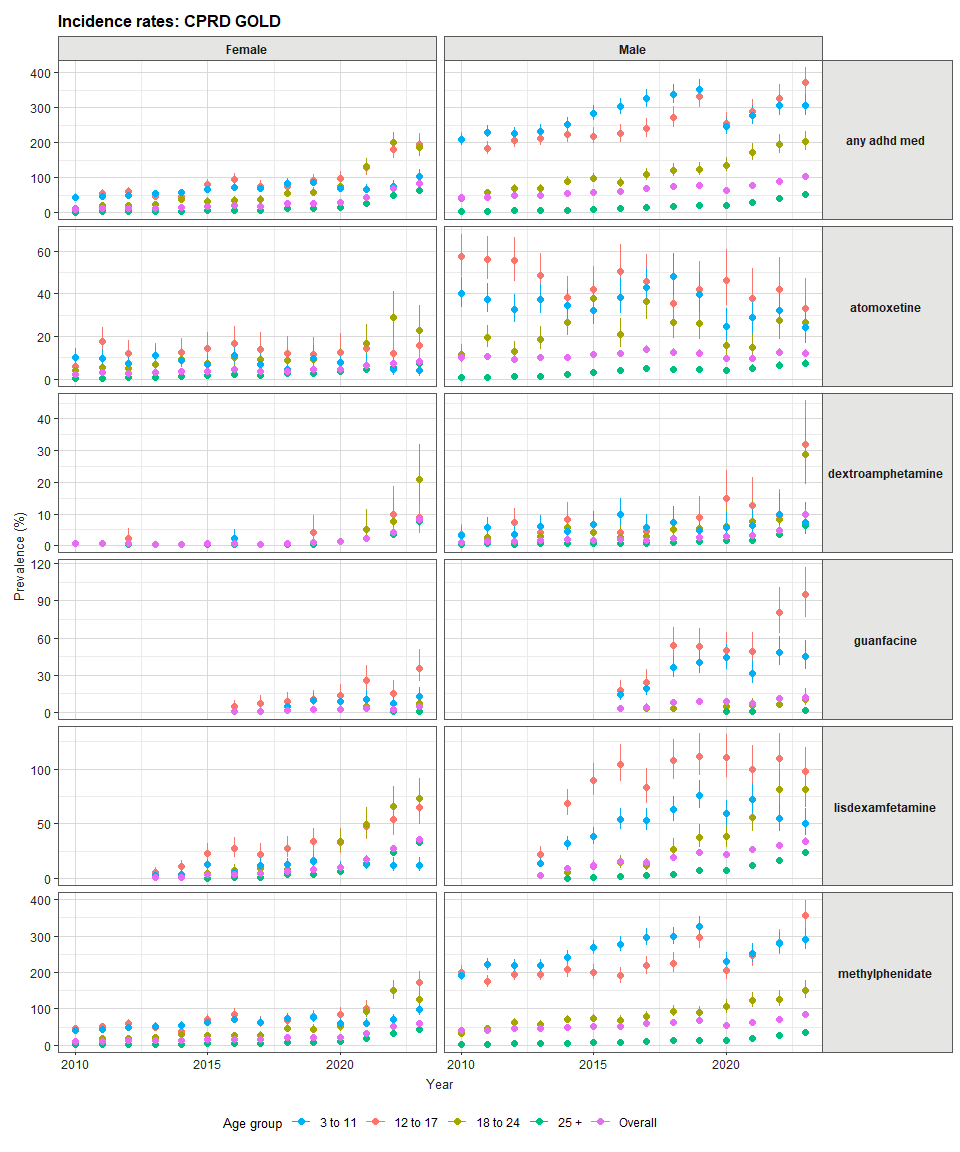


**Figure S14. Age-sex specific PPC**
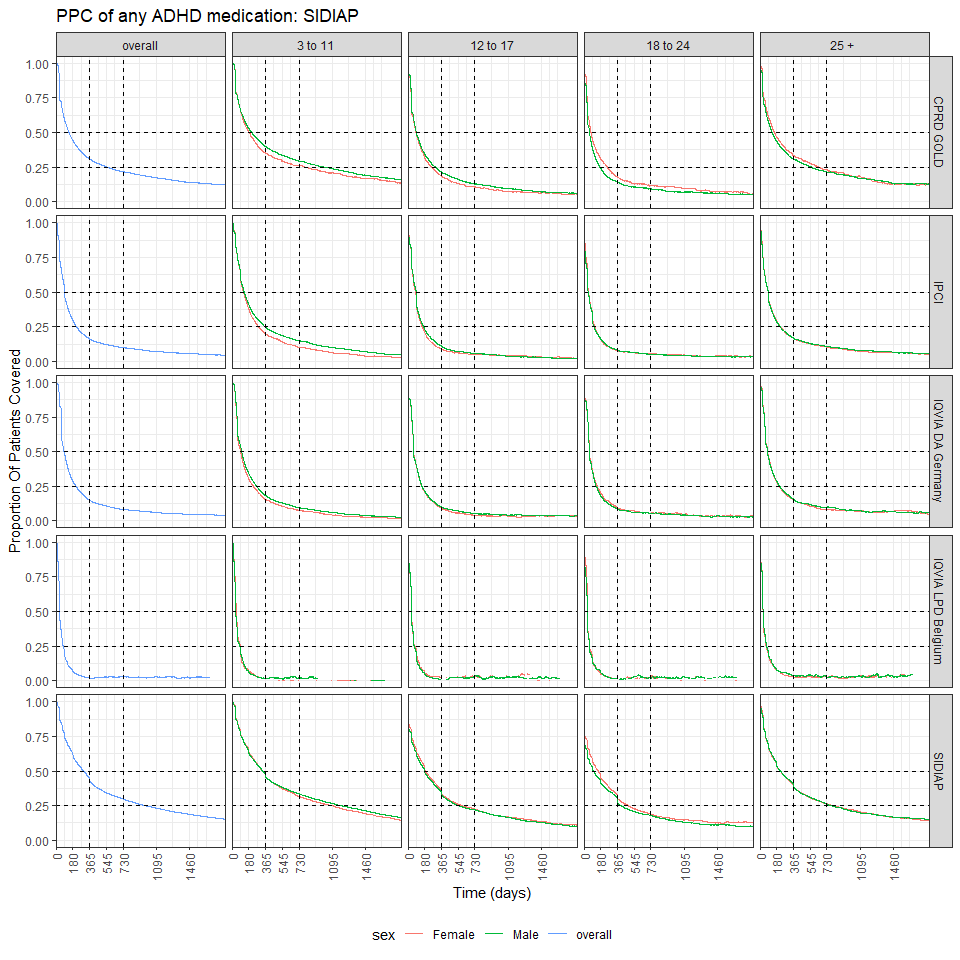


**Table S1. Sensitivity analysis of Proportion of patients covered by ADHD medication after initiation during 5-years follow-up.**

|  | **Germany** | **Netherlands** | **Spain** | **UK** |
| --- | --- | --- | --- | --- |
| **Time** |  |  |  |  |
| **180 days** | 51.4% [50.9% - 51.9%] | 48.0% [47.5% - 48.5%] | 70.3% [70.0% - 70.7%] | 68.0% [67.4% - 68.5%] |
| **1 year** | 36.2% [35.6% - 36.7%] | 33.0% [32.5% - 33.4%] | 53.5% [53.1% - 54.0%] | 56.9% [56.2% - 57.5%] |
| **2 years** | 25.4% [24.8% - 25.9%] | 23.1% [22.7% - 23.6%] | 38.8% [38.4% - 39.2%] | 46.2% [45.5% - 46.9%] |
| **3 years** | 20.9% [20.3% - 21.5%] | 18.5% [18.0% - 19.0%] | 31.1% [30.7% - 31.6%] | 39.9% [39.1% - 40.7%] |
| **4 years** | 17.9% [17.3% - 18.6%] | 15.1% [14.6% - 15.6%] | 25.7% [25.3% - 26.1%] | 35.1% [34.3% - 36.0%] |
| **5 years** | 14.9% [14.2% - 15.6%] | 12.8% [12.3% - 13.3%] | 21.7% [21.3% - 22.1%] | 30.9% [30.0% - 31.9%] |
